# A multiple-perspective approach for the assessment and learning of ultrasound skills

**DOI:** 10.1007/s40037-018-0419-8

**Published:** 2018-04-16

**Authors:** Martin Grønnebæk Tolsgaard

**Affiliations:** Rigshospitalet, Copenhagen Academy for Medical Education (CAMES), Copenhagen, Denmark

**Keywords:** Ultrasound, Mastery learning, Simulation-based medical education, Assessment of performance, Transfer of training, Preparation for future learning

## Abstract

Ultrasound has become a core skill in many specialties. We evaluated the learning and assessment of ultrasound skills in Obstetrics-Gynaecology in a series of eight studies. In the clinical setting, we found that trainees as well as experienced clinicians struggle with technical aspects of performance such as image optimization. We examined how to improve these aspects of performance in the simulated setting by determining mastery learning levels and exploring learning curves for novices. We then examined how to improve the efficiency of training as well as transfer of learning through the use of dyad practice as compared with single practice. We found that the use of simulation-based training focusing on technical aspects of performance in addition to clinical training led to sustained improvements in performance after two months of clinical training in *all* aspects of performance. In addition, we found an interaction effect between initial simulation-based training and subsequent clinical training on trainees’ need for supervision. These findings suggest that simulation-based training can work as preparation for future learning rather than merely as added learning. Finally, we found that the use of simulation-based initial training led to a large decrease in patients’ discomfort, improvements in their perceived safety and confidence in their ultrasound operator. However, simulation-based training comes at a cost and in the final study we developed a model for conducting cost-effectiveness studies and provided data from an example study on how to link training costs with quality of care.

## Introduction

Ultrasound is being used in multiple clinical specialties but we know little about learning and assessment of ultrasound skills. Performing ultrasound examinations relies on fine motor skills as well as visual-cognitive skills. Motor skills are needed for the hand-eye coordination between the ultrasound probe and the ultrasound image. According to the literature on learning motor skills, we may hypothesize that learners initially have difficulties managing hand-eye coordination and that with time their movements become increasingly effortless, smooth, and with fewer errors [1]. Visual-cognitive skills are also essential to interpret the images in order to come to a diagnosis. Research in the development of visual-cognitive skills suggests that as learners become increasingly experienced, they develop more and more sophisticated schemas for the visual presentation of certain ultrasound diagnoses. The process of visual diagnosis is thought to rely on an initial global impression followed by a focal search, where key features are identified in the image and constantly compared against the operator’s previous experiences [2, 3].

In the eight studies [4–11] included in this doctoral dissertation, we examined learning and assessment of ultrasound skills from multiple viewpoints. The studies were informed by multiple theories including research in visual diagnosis, motor skills learning theories, theories from cognitive psychology as well as economic theory.

## Methods

In Study 1 [4], we examined which challenges OB/GYN trainees in Denmark, Norway, and Sweden experienced with ultrasound training using an e‑survey. The survey questions included trainees’ confidence in managing different aspects of the ultrasound examination including their use and attitude toward feedback.

The objective of Study 2 [5] was to develop a generic instrument for the assessment of ultrasound skills and establish international multi-specialty consensus on the contents of this instrument. We wished to collect content evidence for the assessment of ultrasound skills across several specialties and contexts. We included 44 ultrasound experts from Europe, North America, and Australia in a Delphi study, in which we asked them to rate the importance of a number of potential items for an assessment instrument through a number of Delphi rounds.

In Study 3 [6], we aimed at collecting validity evidence for assessments made using the instrument developed in Study 2: The Objective Structured Assessment of Ultrasound Skills (OSAUS). We recorded hand movements and ultrasound output from 30 transabdominal and transvaginal scans performed by OB/GYN trainees and consultants. The resulting videos were assessed by two OB/GYN consultants using the OSAUS scale.

In Study 4 [7], we aimed to collect validity evidence for the assessment of ultrasound skills in the simulated setting and to establish mastery learning levels in order to examine learning curves of ultrasound novices. We included data from 28 OB/GYN trainees and consultants.

In Study 5 [8], we did a non-inferiority randomized study to examine if training in pairs (dyad training) was as effective as training alone (single training) on transfer of learning. A total of 24 participants completed the transfer test. Two raters evaluated pre-test, post-test and transfer-test performances using the OSAUS scale.

In Study 6 [9], we aimed at determining if the immediate effects of initial simulation-based ultrasound training were sustained after two months of clinical ultrasound training. We randomized 33 new OB/GYN trainees to simulation-based mastery learning and clinical training or to clinical training alone. After two months, we recorded one transvaginal ultrasound examination that was evaluated by two OB/GYN consultants using the OSAUS scale.

Study 7 [10] was an extension of Study 6 in terms of evaluating how adding simulation-based training to clinical training impacted trainees’ need for supervised practice, use of time, and patient-reported quality of care over the first 6 months of clinical training. We included data from 52 trainees and from 1,150 patients.

In the final study—Study 8 [11]—we aimed at developing a model for conducting cost-effectiveness analyses in medical education. We did a literature review to inform the model and we included data from an example study on the cost-effectiveness of training midwives in performing cervical examinations.

## Results

In Study 1 a factor analysis revealed that three factors were relevant to trainees’ challenges with performing ultrasound examinations: Technical aspects of performance, image interpretation, and medical management. In particular, trainees ranked their technical skills as low.

The OSAUS scores in Study 2 and 3 provided validity evidence for the assessment of ultrasound skills. Trainees as well as experienced gynaecologists often struggled with technical aspects of performance such as image optimization. The gynaecologists received significantly lower scores than the foetal medicine consultants (Mann Whitney U‑test, *U* = −2.45; *p* = 0.014, Cohen’s *d* = 2.45).

Study 4 provided validity evidence for the assessment of ultrasound skills in the simulated setting. We established mastery learning levels according to the level that a group of foetal medicine consultants performed at. We also included a group of gynaecologists, who performed slightly worse than the foetal medicine consultants (median 88.4% vs. 77.6%, respectively; Mann Whitney U‑test, *p* = 0.05) despite being equally senior in the number of years of clinical experience.

In Study 5, we found that dyad training led to positive effects on transfer of learning with a difference in OSAUS scores of 7.8% (95% CI −3.8 to 19.6%). This difference suggested that dyad training was non-inferior but not superior to single training. However, a significantly larger proportion of dyads than singles passed the pass/fail criterion developed in Study 3 (dyad group, 71.4%; single group 30.0%; Chi-2 = 4.03; *p* < 0.05).

Study 6 demonstrated that trainees who received initial simulation-based training outperformed those who only received clinical training with 20.1 percentage points (95% CI 11.1–29.1) on the OSAUS scale (2 × 2 ANOVA, *p* < 0.001). After having an average of 44 supervised scans in both groups, only 8.3% of the trainees, who only received clinical training, versus 85.7% of trainees in the simulation group were able to pass the pass/fail-criterion established in Study 3.

Study 7 established large improvements in patient-reported safety and a large decrease in patients’ discomfort during transvaginal ultrasound examinations performed by trainees, who received initial simulation-based training compared with those who only received clinical training. Moreover, we found an interaction effect between simulation-based training and subsequent clinical training for the need for supervision or repeat examination. Trainees in the simulation group reduced their odds of needing supervision by 45.3% (95% CI 33.5–55.1) whereas trainees who had not received initial simulation-based training only reduced the odds by 19.8% (CI 4.1–32.9) each time their clinical training time was doubled (log2 transformed data, *p* = 0.005). All regression analyses were conducted using generalized estimating equations (GEE) statistics.

In Study 8, we proposed a four-step model for conducting cost-effectiveness studies in medical education. The model included the following steps: 1) Gathering data on training outcomes 2) Gathering data on training costs 3) Calculating a cost-effectiveness ratio, and 4) Estimating the probability that the intervention is cost-effective.

## Discussion

In the studies outlined above, we used a multi-perspective approach to the assessment and learning of ultrasound skills in Obstetrics-Gynaecology. We included viewpoints from learners and leading experts from multiple countries, and we examined the role of simulation-based ultrasound training from the perspectives of learners, patients, clinician supervisors, and decision-makers.

We found evidence that even experienced clinicians did not display expert behaviour even though they performed ultrasound examinations on a daily basis. This may be because these clinicians took short-cuts in their decision-making because they only needed a minimum of information to reach a diagnosis. However, it may also suggest that when learners are not provided with adequate basic training, they will never attain expert levels of performance through clinical training because they have not acquired the basic skills needed to benefit from their subsequent clinical training. In Study 6 and Study 7 we found further evidence that providing some basic skills may prepare trainees for future learning in ways that cannot be explained by an added effect alone. For example, if the use of simulation-based training only resulted in an added effect on trainees’ performance, we would not have observed an interaction effect between simulation-based training and subsequent clinical training (Study 7).

## Advice

As researchers we need to position our studies between what is of practical value (curriculum development and evaluation) and of general value (developing theory that may be generalized across multiple settings). Hopefully, these two are not opposites but should be combined as this often leads to some of the most important and interesting studies in medical education. However, if we disregard theory in the pursuit of easily-answerable questions or if we become indifferent to the practical implications of our research, then we end up with studies that cannot pass the ‘so what’ test.

## University information

This doctoral thesis was defended at the University of Copenhagen on the 24 November 2017. Opponents: Professors Kevin Eva and Göran Lingman. Mentors: Professors Charlotte Ringsted and Ann Tabor. The full thesis is available at: http://ugeskriftet.dk/files/b5445_assessment_and_learning_of_ultrasound_skills_in_obstetrics_gynecology.pdf.
